# Characterization and Pharmacological Inhibition of the Pore-Forming *Clostridioides difficile* CDTb Toxin

**DOI:** 10.3390/toxins13060390

**Published:** 2021-05-28

**Authors:** Katharina Ernst, Marc Landenberger, Julian Nieland, Katharina Nørgaard, Manfred Frick, Giorgio Fois, Roland Benz, Holger Barth

**Affiliations:** 1 Institute of Pharmacology and Toxicology, Ulm University Medical Center, 89081 Ulm, Germany; marc.landenberger@uni-ulm.de (M.L.); j.nieland@hotmail.de (J.N.); katha.norgaard@gmail.com (K.N.); 2 Institute of General Physiology, Ulm University, 89081 Ulm, Germany; manfred.frick@uni-ulm.de (M.F.); giorgio.fois@uni-ulm.de (G.F.); 3 Department of Life Sciences and Chemistry, Jacobs-University Bremen, 28759 Bremen, Germany; r.benz@jacobs-university.de

**Keywords:** pore-forming toxins, transmembrane pore, *Clostridioides difficile*, bacterial binary AB-toxins, CDT toxin, pore blocker, chloroquine

## Abstract

The clinically highly relevant *Clostridioides* (*C.*) *difficile* releases several AB-type toxins that cause diseases such as diarrhea and pseudomembranous colitis. In addition to the main virulence factors Rho/Ras-glycosylating toxins TcdA and TcdB, hypervirulent strains produce the binary AB-type toxin CDT. CDT consists of two separate proteins. The binding/translocation B-component CDTb facilitates uptake and translocation of the enzyme A-component CDTa to the cytosol of cells. Here, CDTa ADP-ribosylates G-actin, resulting in depolymerization of the actin cytoskeleton. We previously showed that CDTb exhibits cytotoxicity in the absence of CDTa, which is most likely due to pore formation in the cytoplasmic membrane. Here, we further investigated this cytotoxic effect and showed that CDTb impairs CaCo-2 cell viability and leads to redistribution of F-actin without affecting tubulin structures. CDTb was detected at the cytoplasmic membrane in addition to its endosomal localization if CDTb was applied alone. Chloroquine and several of its derivatives, which were previously identified as toxin pore blockers, inhibited intoxication of Vero, HCT116, and CaCo-2 cells by CDTb and CDTb pores in vitro. These results further strengthen pore formation by CDTb in the cytoplasmic membrane as the underlying cytotoxic mechanism and identify pharmacological pore blockers as potent inhibitors of cytotoxicity induced by CDTb and CDTa plus CDTb.

## 1. Introduction

*Clostridioides difficile* (*C. difficile*), formerly known as *Clostridium difficile*, is a clinically highly relevant, Gram-positive anaerobic pathogen that releases several AB-type protein toxins. The large AB-type toxins TcdA and TcdB are considered the main virulence factors which cause the symptoms of *C. difficile* associated disease (CDAD) [1,2,3]. AB-type protein toxins consist of two functional domains. The binding/translocation B-domain mediates binding of the toxin to its cell surface receptor, triggering endocytosis. Then, the B-domains facilitate the translocation of the enzymatically active A-domain from intracellular transport vesicles (e.g., acidified endosomes) into the cytosol. Here, the A-domains modify a specific substrate, which leads to cellular reactions and, thus, to characteristic clinical symptoms [2]. 

The characteristic symptom of CDAD is severe diarrhea that can result in life-threatening complications of pseudomembranous colitis and toxic megacolon. CDAD is highly prevalent in hospitalized patients and in patients at nursing home settings. In most CDAD cases, previous treatment with antibiotics leads to a disturbed gut microbiota, which in turn enables *C. difficile* overgrowth. *C. difficile* releases two large AB-type protein toxins, TcdA and TcdB, which cause glycosylation of Rho/Rac proteins in cells and are considered the major virulence factors of *C. difficile* [4]. However, increasing numbers of hypervirulent strains have been reported that were isolated from more severe CDAD cases. These strains produce a third toxin, the *C. difficile* transferase CDT [5,6], which belongs to the group of binary actin ADP-ribosylating toxins and is closely related to the binary *Clostridium perfringens* (*C. perfringens*) iota toxin [1,7,8,9,10,11]. Although the molecular and cellular effects caused by CDT have been well characterized in recent years, its role as a virulence factor and, therefore, its contribution to disease are still under debate [1,9]. This is due to observations, for example, in a hamster model that *C. difficile* strains only expressing CDT but not TcdA or TcdB do not cause the typical symptoms of CDAD [12,13]. However, CDT might contribute to more severe forms of disease, which have been observed with hypervirulent strains additionally expressing CDT [1,12]. 

CDT consists of two separate protein toxin components: the binding/translocation B-component, CDTb, and an enzymatically active A-component, CDTa. CDTb binds to a specific receptor on the cell surface, the lipolysis-stimulated lipoprotein receptor (LSR). Moreover, CD44 has been identified as a co-receptor [14]. Upon binding of CDTb, LSR clusters in cholesterol-rich microdomains of the cytoplasmic membrane [8,9,15,16]. CDTa binds to CDTb, and the toxin complex is then internalized by receptor-mediated endocytosis. Recently, the 3D structures of CDT were revealed, which show that one CDTa molecule binds to the center of one CDTb heptamer [17]. CDTa is transported by CDTb across the endoplasmic membrane into the cytosol of the target cell [9]. This translocation step is assisted by the host cell chaperones Hsp90, Hsp70, cyclophilins, and FK506 binding proteins [18,19,20,21]. In the cytosol, CDTa covalently transfers an ADP-ribose moiety from NAD^+^ onto its specific substrate, G-actin. F-actin depolymerization, cell rounding, and eventually apoptotic cell death are the consequences [6,22,23,24]. 

We recently reported that the B-component CDTb exhibits cytotoxicity in the absence of CDTa [25]. CDTb alone caused cell rounding in Vero and CaCo-2 cells, as well as a decrease in the cell viability of Vero cells. This cytotoxic effect depends on LSR, the specific cellular receptor of CDTb. Cell rounding was prevented by an enzymatically inactive CDTa mutant or by a cyclodextrin derivative, a known blocker of the transmembrane pores of other binary toxins [25]. Moreover, CDTb caused calcium (Ca^2+^) influx in CaCo-2 cells [26,27]. From in vitro experiments in black lipid bilayers, it is known that CDTb forms pores into membranes [26,28], which serve as translocation channels for the enzymatically active CDTa into the cytosol. A 3D structure analysis revealed distinct states of CDTb from soluble pre-pore to pre-insertion, and partial and then full ß-barrel pore formation at pH 8.0 [29]. Taken together, these results provide evidence that pore formation in the cytoplasmic membrane might be the underlying mechanism of CDTb cytotoxicity. In the present study, we further characterized the cytotoxic CDTb effect in more detail. We demonstrated that treatment of cells with CDTb leads to redistribution of F-actin without causing microtubule-based protrusions that have been reported to be induced by CDTb plus CDTa. Fluorescence-labeled CDTb was detected at least partially at the cytoplasmic membrane in addition to its localization in early endosomes. If CDTb was applied together with CDTa, CDTb signals were predominantly detected in early endosomes and not at the cytoplasmic membrane. Moreover, we showed that chloroquine and several of its derivatives, which were previously identified as pore blockers for binary toxins, protected Vero, HCT 116, and CaCo-2 cells from CDTb intoxication and inhibited CDTb pores in vitro and in cells. Furthermore, chloroquine also inhibited cytotoxic effects of the combination of CDTa plus CDTb. 

## 2. Results

### 2.1. CDTb Impairs Cell Viability of CaCo-2 Cells

**Figure 1:**
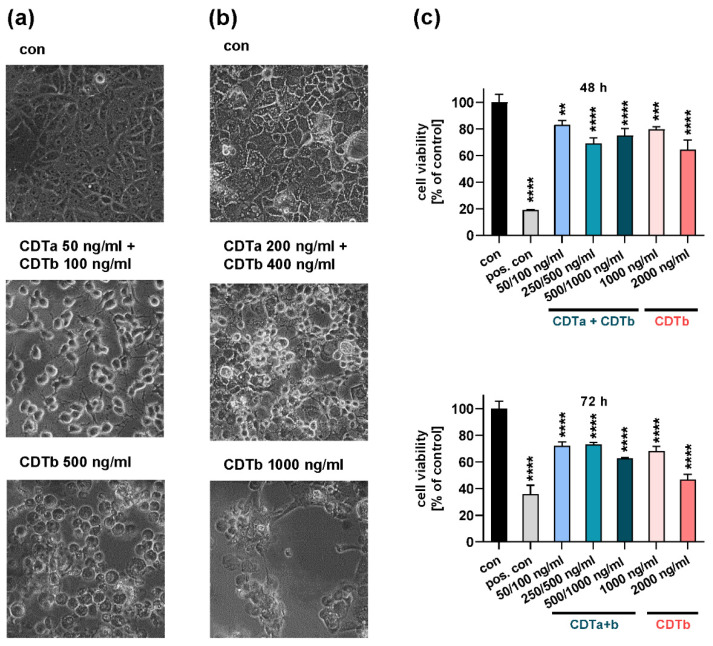
CDTb causes cell rounding and impairs cell viability. Vero (**a**) and CaCo-2 (**b**) cells were incubated with a combination of CDTa and CDTb or with CDTb alone. After 3 h, images of cell morphology were taken. (**c**) Cell viability of CaCo-2 cells was determined after 48 and 72 h after incubation with different concentrations of CDTa plus CDTb or CDTb alone. For positive control (pos. con), cells were exposed to osmotic shock by double-distilled water. Values are given as percentage of control, mean ± SD (*n* = 3). Significance was tested using one-way ANOVA followed by Dunnett’s multiple comparison test (** *p* ≤ 0.01, *** *p* ≤ 0.001, **** *p* ≤ 0.0001 vs. con (untreated control).

As demonstrated before [25], CDTb causes cell rounding in Vero and CaCo-2 cells in the absence of CDTa. Rounding of cells occurs at relatively high concentrations, early after treatment with CDTb, and is morphologically distinct from cell rounding induced by the combination of CDTa and CDTb (Figure 1). Here, we additionally showed that CDTb impairs cell viability of CaCo-2 cells after 48 and 72 h (Figure 1c). This effect was more pronounced with the higher concentration of CDTb. 

### 2.2. CDTb Leads to Redistribution of F-Actin in CaCo-2 cells

**Figure 2:**
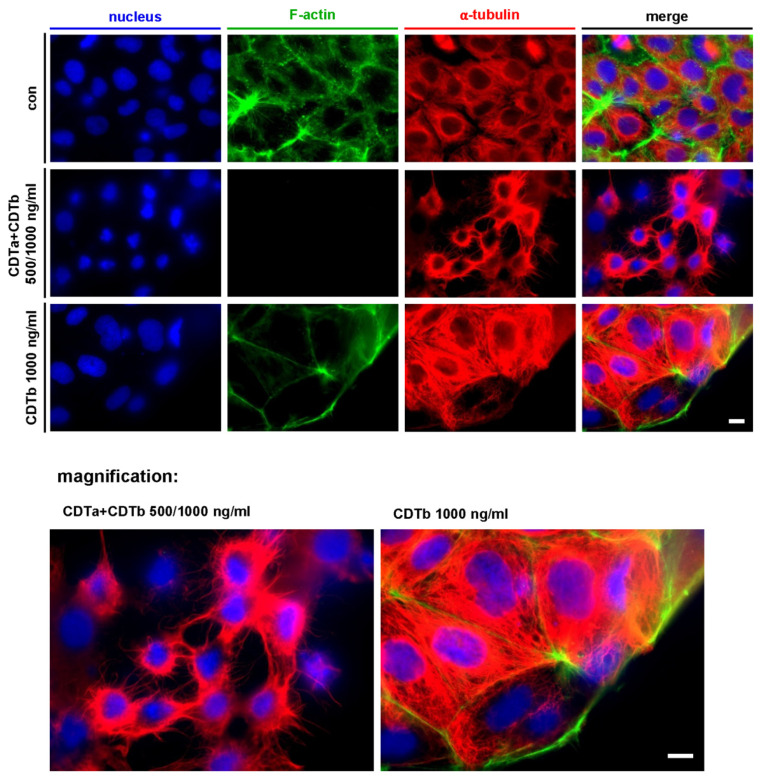
CDTb does not induce microtubule-based protrusions. CaCo-2 cells were incubated for 1 h with CDTa plus CDTb or with CDTb alone. For control, cells were left untreated. Cells were fixed, permeabilized, and after blocking, nuclei (by Hoechst 33342), F-actin (by phalloidin-FITC), and microtubules (by mouse anti-α-tubulin and Alexa568 goat anti-mouse antibodies) were visualized. Scale bar = 10 µm.

Treatment of CaCo-2 cells with the combination of CDTa plus CDTb led to the typical loss of F-actin (Figure 2). G-actin that has been ADP-ribosylated by CDTa acts as a capping protein on actin filaments, inhibiting the addition of further G-actin monomers. This leads to depolymerization of the actin cytoskeleton and loss of F-actin signal (Figure 2, second row). Moreover, the formation of microtubule-based protrusions (Figure 2, second row and magnification) was induced by CDTa plus CDTb as described before [30,31]. In contrast, CDTb did not cause microtubule-based protrusions but led to the redistribution of F-actin signals in cells treated with CDTb alone (Figure 2, third row). The signal for F-actin is reduced in the cell body and is concentrated at the cell cortex. The increased signal for α-tubulin in CDTb-treated cells is most likely due to cells starting to round up, causing a condensed and thus intensified signal. 

### 2.3. CDTb Is Partially Localized at the Cytoplasmic Membrane

**Figure 3:**
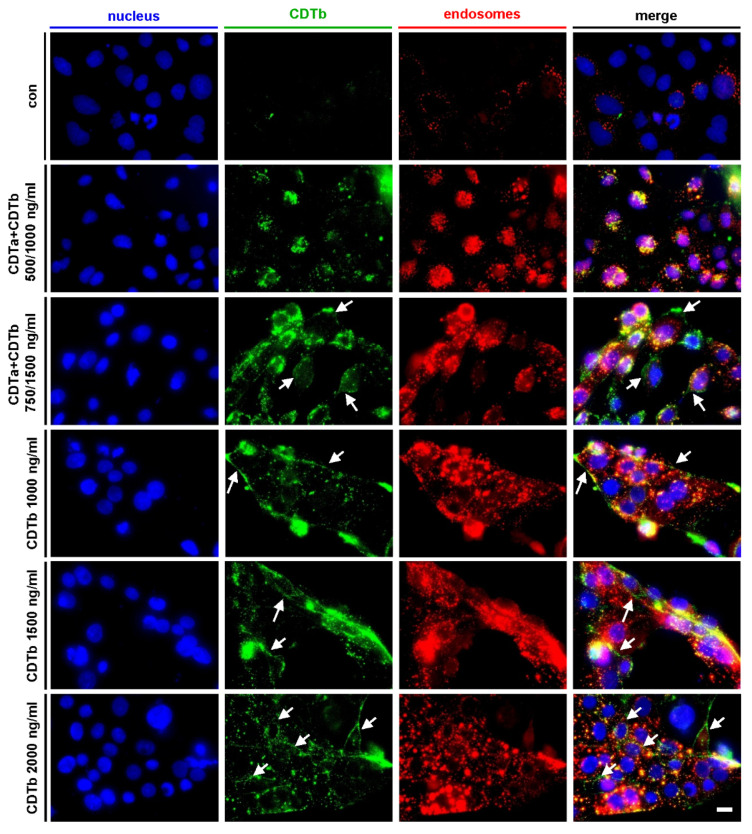
CDTb is localized at the cytoplasmic membrane. CaCo-2 cells were incubated for 1 h with DyLight488-labeled CDTb in the presence or absence of unlabeled CDTa. For control, cells were left untreated. After fixing, permeabilization, and blocking, nuclei (by Hoechst 33342) and early endosomes (by goat anti-EEA1 and mouse anti-goat-CFL647 antibodies) were visualized additionally to DyLight488-labeled CDTb. Z-stacks were taken, and two-dimensional Z-projections were generated with ImageJ. Arrows indicate CDTb signals at cell boundaries, i.e., cytoplasmic membrane. Scale bar = 10 µm.

When cells are treated with 500 ng/mL CDTa plus 1000 ng/mL CDTb (Figure 3, second row), CDTb signals are predominantly found as dots that colocalize with signals of early endosomes. This indicates that the majority of CDTb is endocytosed if applied together with CDTa. When cells are exposed to the same concentration of CDTb (1000 ng/mL) in the absence of CDTa (Figure 3, 4th row), CDTb signals at the cell borders that did not colocalize with early endosomes are detected additionally to endosomal CDTb. This distribution of CDTb signal is also found in higher concentrations of CDTb (Figure 3, 5th and 6th row). Moreover, CDTb signals at cell borders are also observed if higher concentrations of CDTa plus CDTb are applied (Figure 3, 3rd row). These results indicate that CDTb alone and in higher concentrations, also in the presence of CDTa, is only partially endocytosed and partially resides at the cytoplasmic membrane. This is in line with our earlier finding that CDTb forms pores into the cytoplasmic membrane, thereby causing cytotoxicity [25,26,27]. 

### 2.4. Chloroquine and Chloroquine Derivatives Protect Cells from CDTb Cytotoxicity

Chloroquine and chloroquine derivatives have been described before as inhibitors of *Bacillus anthracis* anthrax toxins, *C. botulinum* C2 toxin, and *C. perfringens* iota toxin by blocking the translocation pore formed by the toxins’ respective B-components [32,33,34,35,36,37,38,39]. Moreover, it was shown that chloroquine blocks CDTb pores in lipid bilayer membranes [28]. Here, we investigated the effect of chloroquine and members of two chloroquine derivative families, chloroquine-related heterocyclic fused azinium salts (azolopyridinium salts, HA1383, HA1495, HA1568), and 4-aminoquinolines (quinacrine) on the cytotoxic effect of CDTb. 

**Figure 4:**
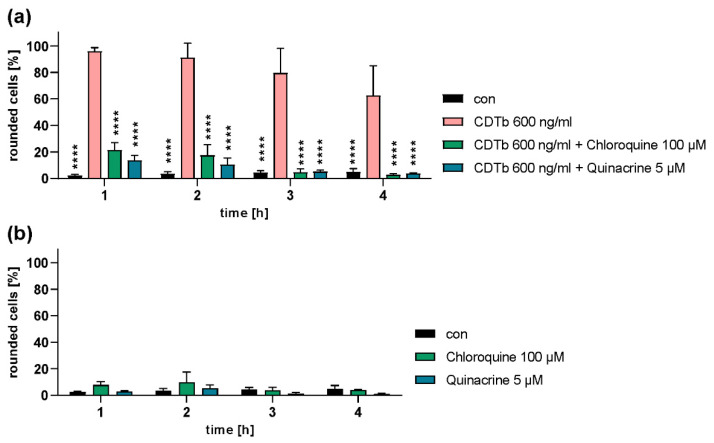
Cells are protected from CDTb-induced cell rounding by chloroquine and quinacrine. Vero cells were treated with CDTb in the presence or absence of chloroquine and its derivative quinacrine at indicated concentrations (**a**). For control, cells were treated with chloroquine and quinacrine alone (**b**). For further control, cells were left untreated (con). Images were taken after the indicated time points, and percentage of rounded cells was determined. Values are given as percentage of control, mean ± SD (*n* = 3). Significance was tested by two-way ANOVA followed by Dunnett’s multiple comparison test. (**a**) All samples were compared to samples treated with CDTb only. **** *p* ≤ 0.0001. (**b**) No significant differences were detected between all samples.

Chloroquine or its derivative quinacrine were applied to Vero cells together with CDTb and inhibited CDTb-induced cell rounding (Figure 4a). Notably, the inhibitors alone had no effect on cell morphology (Figure 4b). 

**Figure 5:**
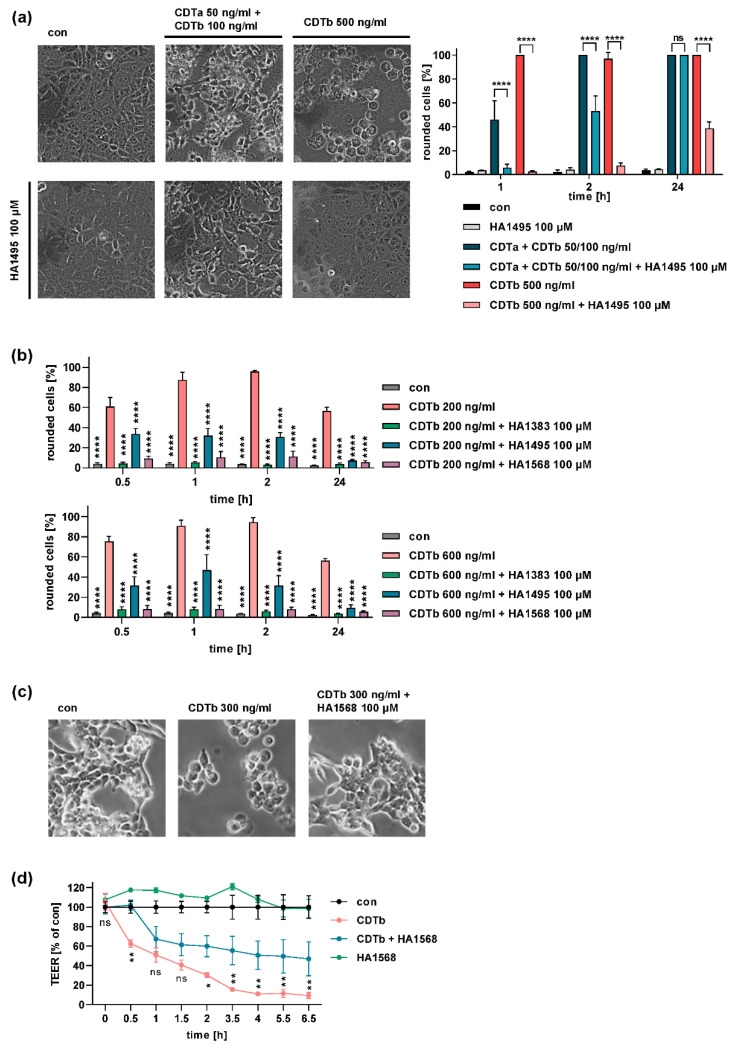
The intoxication of cells by CDTb is reduced by azolopyridinium salts. (**a**) Vero cells were treated with the combination of CDTa plus CDTb or with CDTb alone in the presence or absence of HA1495. For control, cells were left untreated (con) or treated with HA1495 alone. Images show cell morphology after 3 h of incubation with toxins and the inhibitor. Percentages of rounded cells were determined from images after the indicated time points. Values are given as percentage of control, mean ± SD (*n* = 3). Significance was tested by two-way ANOVA followed by Dunnett’s multiple comparison test. (**b**) Vero cells were incubated with two different concentrations of CDTb in the presence or absence of three different azolopyridinium salts. Analysis was performed as described in (**a**). Significance was tested by two-way ANOVA followed by Dunnett’s multiple comparison test vs. samples treated with CDTb alone. (**c**) HCT116 cells were incubated with CDTb in the presence or absence of HA1568. For control, cells were left untreated (con). Images were taken after incubation for 1 h. (**d**) CaCo-2 cells were seeded on filter inserts and treated with 500 ng/mL CDTb in the presence or absence of 100 µM HA1568. For control, cells were left untreated (con) or were treated with HA1568 only. The transepithelial electrical resistance was measured at the indicated time points. Values were normalized to untreated control and are given as mean ± SD (*n* = 4). Significance between samples treated with CDTb only and CDTb plus HA1568 was tested by two-way ANOVA followed by Dunnett’s multiple comparison test. (* *p* ≤ 0.05, ** *p* ≤ 0.01, **** *p* ≤ 0.0001, ns = not significant).

The azolopyridinium salts HA1383, HA1495, and HA1568 protected cells from intoxication with CDTb (Figure 5). Vero cells treated with CDTb together with the chloroquine derivative HA1495 were almost completely protected from CDTb-induced cell rounding. Only after longer incubation periods of 24 h did cells round up in the presence of the chloroquine derivative (Figure 5a). Moreover, the chloroquine derivative also protected cells from the combination of CDTa plus CDTb. However, the protective effect was more pronounced against CDTb alone (Figure 5a). Two other structurally similar chloroquine derivatives, HA1383 and HA1568, also inhibited CDTb-induced cell rounding (Figure 5b). Not only Vero cells but also two human colon epithelial cell lines, HCT 116 and CaCo-2 cells, were protected from CDTb cytotoxicity by a chloroquine derivative (Figure 5b,c). Fewer rounded HCT 116 cells were observed if cells were treated with CDTb in the presence of the chloroquine derivative (Figure 5c). Moreover, the chloroquine derivative delayed the CDTb-induced loss of epithelial barrier of CaCo-2 cells determined by measuring the transepithelial electrical resistance (TEER) of a CaCo-2 monolayer (Figure 5d). TEER measurements represent a more sensitive endpoint than cell rounding because effects on cell–cell contacts, such as tight junctions that are not visible in light microscopy, also cause a decrease in transepithelial resistance. Therefore, a CDTb concentration of 500 ng/mL was used in this experiment, which caused only mild morphological impairments observed in light microscopy [25]. 

### 2.5. Chloroquine Blocks CDTb Pores In Vitro

**Figure 6:**
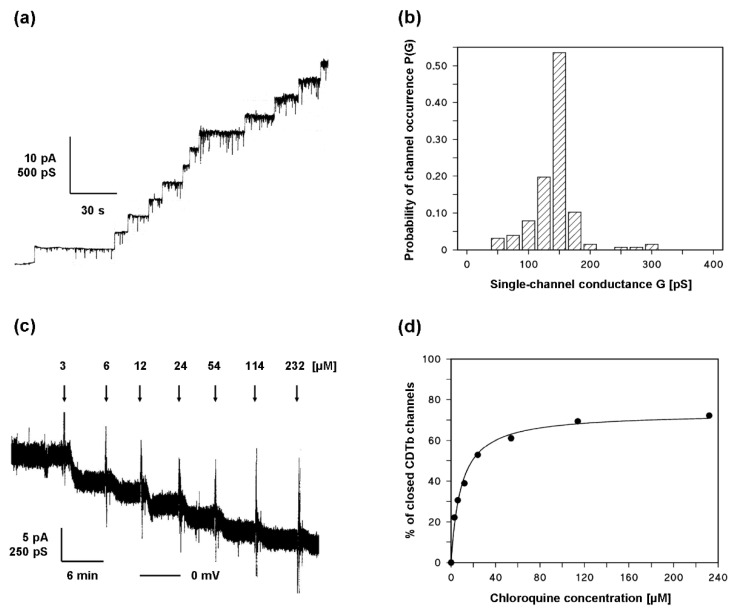
Chloroquine inhibits CDTb pores in lipid bilayers. (**a**) Single-channel recording of a diphytanoyl phosphatidylcholine/n-decane membrane in the presence of 160 ng/mL CDTb added to the cis side of a black membrane. The aqueous phase contained 1 M KCl, 10 mM MES, pH6. The applied membrane potential was 20 mV; T = 20 °C. (**b**) Histogram of the probability P(G) of occurrence of a given conductivity unit observed with membranes formed of PC/n-decane in the presence of CDTb taken from experiments similar to the conductance steps in Figure 6a. P(G) is Table (147 ± 16) pS for 127 conductance steps. (**c**) Titration experiment of CDTb-induced membrane conductance with chloroquine. The membrane was formed from diphytanoyl phosphatidylcholine/n-decane. The aqueous phase contained 2 nM CDTb (added to the cis side of the membrane), 100 mM KCl, 10 mM MES, pH 6.0, and chloroquine added in the concentrations shown on the top of the figure to both sides of the membrane. The temperature was constant at 20 °C, and the applied voltage was 20 mV. The membrane contained about 25 CDTb channels. The bottom line represents zero level of conductance. (**d**) Langmuir adsorption isotherm of the inhibition of CDTb-induced membrane conductance by chloroquine. The data taken from Figure 6b were fitted to Equation (3). The inhibition constant, K, for a chloroquine-induced channel block was (1.1 × 10^5^ ± 0.02 × 10^5^) 1/M. The block was at maximum 74% ± 1.8%; half saturation constant K_S_ = 9.1 µM (r^2^ = 0.9929).

The addition of small amounts of CDTb in nanomolar concentration led to a strong increase in membrane conductance caused by channel insertion in the membrane. Figure 6a shows the channel formation by CDTb in artificial bilayers in 1 M KCl solution. The channels showed some flickers to lower conductance states but otherwise had a stepwise appearance with a long lifetime. The single-channel conductance was around 150 pS under these conditions (see Figure 6b). When the reconstitution of CDTb channels was followed for a longer time, the conductance was virtually stationary after about 30 to 60 min. At that time, the titration of membrane conductance with chloroquine started by adding small amounts of concentrated chloroquine solution to both sides of the membrane while stirring. Shortly after each addition, the CDTb-induced conductance decreased in a dose-dependent manner as shown in Figure 6c. The titration curve given in Figure 6c can be analyzed using a Langmuir adsorption isotherm, as shown in Figure 6d. The analysis yielded a stability constant K of 110,000 M^−1^ (half saturation constant K_s_ of 9.1 µM) for the binding of chloroquine to the CDTb channels. The percentage of conductance that responded to ligand binding was about 74% in the case of the experiment shown in Figure 6c,d.

### 2.6. A Chloroquine Derivative Prevents Ca^2+^ Influx through CDTb Pores in Cells

**Figure 7:**
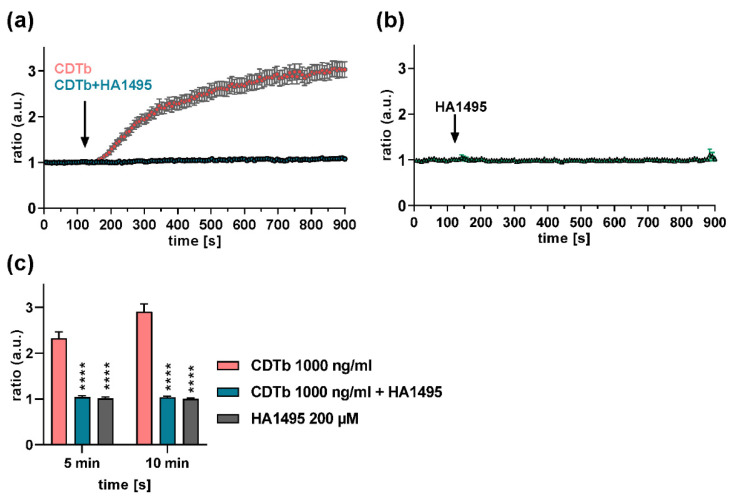
HA1495 prevents CDTb-mediated Ca^2+^ influx in CaCo-2 cells. CaCo-2 cells were incubated with Fura-2 (3 µM) for 45 min at 37 °C. After baseline recording for 2 min of the ratio of Fura-2 at 340 and 380 nm, 1000 ng/mL CDTb or 1000 ng/mL CDTb plus 200 µM HA1495 were added to the cells simultaneously (**a**). For control, cells were treated with 200 µM HA1495 alone (**b**). Time of applications is indicated by arrows. (**c**) Bar graph showing results 5 min and 10 min after initial application of different treatments (first arrow in a, b). Values were normalized to untreated control and are given as mean ± SD (*n* = 3). Significance vs. CDTb was tested by two-way ANOVA followed by Dunnett’s multiple comparison test. (**** *p* ≤ 0.0001).

We reported earlier that CDTb forms Ca^2+^ permeable pores in the cytoplasmic membrane of CaCo-2 cells [26,27]. Here, we demonstrate that CDTb-mediated Ca^2+^ influx was inhibited by the chloroquine derivative HA1495 (Figure 7). Quantification of Ca^2+^ influx 5 and 10 min after application of CDTb together with the chloroquine derivative also showed a significant inhibition of CDT-induced Ca^2+^ influx by the chloroquine derivative (Figure 7c). Notably, the chloroquine derivative alone had no effect on intracellular Ca^2+^ levels (Figure 7b,c).

These taken together, we extended the current knowledge on the cytotoxic effect of CDTb and demonstrated that CDTb leads to a redistribution of F-actin from the cell body to the cellular cortex. Microtubule-based protrusions were not induced by CDTb alone. Moreover, fluorescence-labeled CDTb was at least partially detected at the cell membrane additionally to its localization in early endosomes. In combination with CDTa, CDTb was predominantly colocalized with early endosomes. Chloroquine and its derivatives efficiently protected Vero and human HCT 116 as well as CaCo-2 cells from intoxication with CDTb and inhibited CDTb pores in vitro and in cells. Moreover, a chloroquine derivative also inhibited intoxication of cells with the combination of CDTa plus CDTb. Our results further establish a possible role of the binding component CDTb of the binary CDT toxin as a virulence factor of hypervirulent *C. difficile* strains and revealed chloroquine and its derivatives as potent inhibitors of CDTb cytotoxicity. 

## 3. Discussion

Bacterial AB-type toxins are characterized by their unique structural and functional organization. The A-subunit of these toxins harbors enzymatic activity and is transported into the cytosol by the B-subunit [7,40]. Therefore, the B-subunit binds to a specific receptor on the cell surface, which triggers endocytosis of the AB-complex. Subsequently, the B-subunit mediates translocation of the A-subunit across a cellular barrier into the cytosol, where the A-subunit modifies a specific substrate. This causes cellular effects and, thus, specific clinical symptoms of the respective toxin-associated diseases. The cytotoxic effect of AB-type toxins depends on the specific activities of both functional subunits. In the case of binary clostridial toxins, to which CDT belongs, translocation of the A-component into the cytosol is mediated by the B-component via pore formation into the endosomal membrane. This pore formation is triggered by acidification of the endosomal lumen [9,24]. 

We recently reported that CDT deviates from this established AB-type model and showed that the B-component CDTb causes cytotoxicity in the absence of its respective enzyme component CDTa [25]. Here, we extended these findings and investigated the cellular uptake of CDTb and its effects on the cytoskeleton by fluorescence microscopy. In combination with CDTa, CDTb was detected mainly in early endosomes. If the concentration of CDTa and CDTb was increased or if CDTb was applied in the absence of CDTa, CDTb signals were also found at the cytoplasmic membrane. These results are in line with our finding that CDTb forms pores into the cytoplasmic membrane and thus exerts its cytotoxic effect. 

Interestingly, the intracellular trafficking of Ib, the B-component of *Clostridium perfringens* iota toxin, was previously investigated, revealing that Ib was also taken up into early endosomes in the absence of its respective enzyme component Ia [41]. At later time points, Ib was found in late endosomes and then lysosomes, and degraded Ib was transported back to the cytoplasmic membrane [41]. Iota toxin and CDT display some similarities regarding their cellular uptake [7,40]. Both toxins require LSR as their cellular receptor [15]. Moreover, iota toxin and CDT share sequence homology to a degree that their A- and B-components are interchangeable [7]. In the same study by Nagahama et al., it was shown that Ib causes Ca^2+^ influx into MDCK cells in a concentration-dependent manner [41]. Interestingly, pore formation by Ib was not investigated, but it was reported that Ib causes cytotoxicity by inducing rapid necrosis in two epithelial cell lines, but not in other cell lines such as Vero or CaCo-2 cells [42]. Moreover, we previously showed that Ib causes cytotoxic effects in Vero cells similar to CDTb [43]. In the present study, we demonstrated that CDTb causes Ca^2+^ influx into CaCo-2 cells and pore formation in black lipid bilayers, which was both inhibited by a known pore blocker. Therefore, we further established the hypothesis that pore formation in the cytoplasmic membrane is the underlying cytotoxic mechanism of CDTb. These commonalities of Ib and CDTb effects suggest that pore formation might be a common cytotoxic mechanism for the group of iota-like toxins. 

Recently, the crystal structure of oligomeric CDTb structures has been described in two studies [29,44]. Interestingly, both studies discovered di-heptameric structures of CDTb as an oligomeric state. Comparison with the structure of protective antigen, the pore-forming component of anthrax toxin, revealed significant differences. Most importantly, an additional receptor-binding domain of CDTb was found that was not present in protective antigen [29,44]. This domain likely contributes to the stabilization of CDTb oligomers prior to receptor binding and membrane insertion through the shielding of several hydrophobic residues [44]. Anderson et al. investigated pore formation and membrane insertion of CDTb in more detail and observed that all structural states from pre-pore to pre-insertion to partial-ß-barrel to pore were detected at pH 8.0 [29]. This suggests that receptor binding might be more important for pore formation than low pH in acidified endosomes, which would be in line with our finding that CDTb forms pores at the cytoplasmic membrane. Although the biological role of the CDTb di-heptamers is still elusive, the authors hypothesize that it might advance membrane insertion. Moreover, the additional heptamer might be important to protect and stabilize the pore-forming CDTb, thereby possibly increasing its in vivo half-life [44]. These special structural characteristics of CDTb oligomer formation that differ from well-studied related anthrax protective antigen might also play a role in the cytotoxic pore-forming mechanism of CDTb described here. 

Fluorescence experiments also revealed that F-actin was redistributed in CDTb-treated cells. The F-actin signal in the cell body was decreased compared to untreated controls, and mainly cortical F-actin was detected. It is known that the combination of CDTa plus CDTb causes a loss of F-actin due to the ADP-ribosylation of G-actin by CDTa [5,6]. However, the mechanism by which CDTb causes redistribution of F-actin in the absence of CDTa is not known and might also be a secondary effect due to osmotic changes caused by CDTb-induced pore formation. CDTb did not induce the formation of microtubule-based protrusions like it was shown for the combination of CDTa plus CDTb. This is in line with the finding that this effect was dependent on ADP-ribosylation of G-actin by CDTa and protrusions preferably occurring at spots of the cytoplasmic membrane where cortical F-actin was reduced [9,30,31].

Although the large Rho/Ras-glycosylating toxins TcdA and TcdB are regarded as the main virulence factors of *C. difficile*, increasing numbers of strains have been identified that additionally release CDT [1,9,45,46]. The role of CDT as a virulence factor is still under debate. It was shown that microtubule-based protrusions induced by CDT lead to an increased adherence of *C. difficile* bacteria in cell culture experiments and also in the mouse model of *C. difficile* infection [30]. Moreover, CDT was shown to cause increased inflammation and suppress protective colonic eosinophilia [47]. These effects depended on the enzyme activity exerted by CDT. Our finding that CDTb has pore-forming activity and thereby harbors cytotoxicity in the absence of CDTa might also be a relevant mechanism of CDT as a virulence factor. *C. difficile* strains only expressing CDT but not TcdA or TcdB are considered enterotoxic, and cases with enterotoxic symptoms such as diarrhea have been reported previously [48]. However, these symptoms differed from typical TcdA/TcdB-induced symptoms, and in most described cases or animal studies, the course of the disease was milder compared to infection where TcdA/TcdB were present [1,12,13]. Recently, a *C. difficile* strain expressing CDT but not TcdA or TcdB was isolated from a patient suffering from several recurrent infections and colonizations. Although this patient also suffered from end-stage chronic kidney disease and congestive heart failure, *C. difficile* infection was likely contributing to the fatal outcome [46]. This and other reports strengthen the importance of CDT as a contributing virulence factor that should be pharmacologically targeted [49]. In the present study, we showed that chloroquine and its derivative efficiently protect cells from intoxication with CDTb by inhibiting CDTb pores in cytoplasmic membranes. Moreover, the same derivative inhibited the intoxication of cells with the combination of CDTa plus CDTb, demonstrating that one inhibitor is able to protect cells from both cytotoxic mechanisms. Previous studies showed that chloroquine blocked CDTb pores in vitro in black lipid bilayers and that chloroquine and several derivatives also blocked the pores of C2 toxin, iota toxin, and anthrax toxin in vitro and in cells [28,32,33,34,35,36,37,38,39].

These taken together, we characterized the cytotoxic effect of CDTb in more detail and showed that CDTb causes impairment of CaCo-2 cell viability as well as redistribution of F-actin without leading to microtubule-based protrusions. Chloroquine and its several derivatives prevented CDTb as well as CDTa plus CDTb induced cytotoxicity. The findings support our hypothesis that CDTb represents a pore-forming toxin in the absence of CDTa and suggest that pore blockers might be attractive starting points for the development of novel therapeutic strategies directly targeting at the toxin level in the context of CDAD. This might complement current antibiotic therapy as well as neutralizing antibodies against TcdB. 

## 4. Materials and Methods

### 4.1. Protein Expression and Purification

CDTa and CDTb were purified and activated as recombinant proteins as described before [50]. For fluorescence microscopy experiments, CDTb was labeled with DyLight488 in accordance with the manufacturer’s recommendations (Thermo Fisher Scientific, Waltham, MA, USA). Micro Bio-Spin 6 columns (Bio-Rad laboratories, Munich, Germany) were used to remove excess dye. 

### 4.2. Cell Culture and Intoxication Experiments

Vero cells (African green monkey kidney cells, DSMZ, Braunschweig, Germany), HCT 116 (human colon carcinoma cells, DSMZ), and CaCo-2 (human epithelial colorectal adenocarcinoma cells, ATCC HTB-37, Manassas, VA, USA) were cultivated as described before [25]. 

For intoxication experiments, cells were seeded in culture dishes, and toxin components were added. Well plates and culture dishes were purchased from TPP Techno Plastic Products (Trasadingen, Switzerland). To test the effect of inhibitors, the respective inhibitor was added to the cells together with the toxin components. After different incubation times, images were taken using a Zeiss (Oberkochen, Germany) Axiovert 40CFL microscope with a Jenoptik (Jena, Germany) ProGres C10 CCD camera. The percentage of rounded cells was determined from the images using ImageJ (NIH, Bethesda, MD, USA). Chloroquine and quinacrine were obtained from Sigma Aldrich (Munich, Germany) and were dissolved in H_2_O. HA1383, HA1495, and HA1568 were synthesized as described before [32,51,52,53,54,55]. 

### 4.3. Cell Viability

Cell viability was measured via MTS assay (Promega, Mannheim, Germany). Therefore, cells were seeded in 96-well plates, and toxin components were added for indicated times. MTS reagent was added, and after 1 h, absorbance at 490 nm was measured. 

### 4.4. Fluorescence Microscopy

Cells were seeded in 8-well plates (ibidi, Martinsried, Planegg, Germany). After treatment with toxin components, cells were washed with PBS and fixed with PFA for 15 min. After washing with PBS, cells were permeabilized with Triton X-100, and autofluorescence was quenched with glycine. Then, cells were blocked in 5% milk powder in PBST and incubated with primary antibodies (mouse anti-α-tubulin (Santa Cruz Biotechnology, Dallas, TX, USA), goat anti-EEA1 (early endosome marker, Sicgen, Cantanhede, Portugal)), and after washing with PBST with secondary antibodies (goat anti-mouse-568 (Thermo Fisher Scientific, Waltham, MA, USA), mouse anti-goat-647 (Santa Cruz Biotechnology)). F-actin was stained with Phalloidin-FITC (Merck, Sigma-Alrich) and nuclei with Hoechst 33,342 (Thermo Fisher Scientific). Images were obtained with an iMic Digital Microscope using the Live Acquisition 2.6 software (FEI, Munich, Germany) and were processed with ImageJ. 

### 4.5. TEER Measurements

TEER measurements were performed as described before [25]. Values of control samples were set to 100%, and other values were normalized to control for every time point. 

### 4.6. Lipid Bilayer Experiments 

The method used for the reconstitution experiments by the black lipid bilayer membranes has been described previously [38]. The membranes were formed from a 1% (*w*/*v*) solution of diphytanoyl phosphatidylcholine (PC) (Avanti Polar Lipids, Alabaster, AL, USA) in n-decane. The membrane current was measured with a pair of Ag/AgCl electrodes switched in series with a voltage source and a highly sensitive current amplifier (Keithley 427). The amplified signal was monitored with a digital oscilloscope and recorded using a strip chart recorder (Rikadenki, Freiburg, Germany). 

The binding of chloroquine to the CDTb channel was investigated with titration experiments similar to those performed previously to study the binding of 4-aminoquinolones to the C2II and PA_63_ channels in multichannel experiments [36,56,57]. The CDTb channels were reconstituted into lipid bilayers. About 60 min after the addition of activated CDTb to the cis side of the membrane, the rate of channel insertion in the membranes was very small. Then small amounts of concentrated solutions of chloroquine were added to the cis side of the membranes while stirring to allow equilibration. The results of the titration experiments, i.e., the blockage of the channels, i.e., the decrease in the conductance as a function of the chloroquine concentration, were analyzed using Langmuir adsorption isotherms (see Equation (1); [56,58]). The conductance, *G*(*c*), of a CDTb channel with stability constant *K* and chloroquine concentration *c* is given by the maximum conductance (without ligand), *G_max_*, times the probability that the binding site is free:

(1) $$G\left(c\right)=\frac{G_{max}}{\left(1+K\cdot c\right)}$$

The half saturation constant *K_s_* is given by the inverse stability constant 1/*K*. Equation (1) can also be rewritten in the following from (i.e., in the form of Langmuir adsorption isotherm [59]:

(2) $$\frac{G_{max}-G\left(c\right)}{G_{max}}=\frac{K\cdot c}{\left(K\cdot c+1\right)}$$

In some cases, not all reconstituted CDTb channels responded to the presence of the channel blocker chloroquine. In these cases, Equation (2) had to be modified to account for this problem:

(3) $$\frac{G_{max}-G\left(c\right)}{G_{max}}=A\cdot \frac{K\cdot c}{\left(K\cdot c+1\right)}$$

A is the fraction of the total number of CDTb channels that responded to the presence of chloroquine in the aqueous phase (normally around 0.7 to 1.0).

### 4.7. Calcium (Ca^2+^) Imaging

Calcium imaging was done as previously described [26]. In brief, cells were incubated in bath solution and loaded with 3 µM Fura-2AM for 45 min at 37 °C. After washing with bath solution, baseline was measured for 2 min before respective treatments. Calcium flow was recorded for 20 min using an iMic Digital Microscope and Live Acquisition 2.6 software.

### 4.8. Reproducibility of Experiments and Statistics

All experiments were performed independently at least three times. In each experiment, triplicates were performed. Figures display one representative result of one experiment with triplicates unless indicated otherwise. Significance was tested by one-way or two-way ANOVA followed by Dunnett’s multiple comparison test. 

## Data Availability

The datasets generated and/or analyzed during the current study are either included in the study or available from the corresponding author on reasonable request.
